# Effects of crude oil and high salinity on eggs and early naupliar stages of the copepod *Calanus hyperboreus*

**DOI:** 10.1093/plankt/fbaf053

**Published:** 2025-09-28

**Authors:** Iliana Vasiliki Ntinou, Sinja Rist, Sofie Rask, Martin Lindegren, Torkel Gissel Nielsen, Øystein Varpe

**Affiliations:** Department of Biological Sciences, University of Bergen, Thormøhlensgate 53B, P.O. Box 7803, NO-5020 Bergen, Norway; Bjerknes Centre for Climate Research, Jahnebakken 5, P.O. Box 7800, NO-5020 Bergen, Norway; National Institute of Aquatic Resources, Technical University of Denmark, Kemitorvet Bygning 202, DK-2800 Kongens Lyngby, Denmark; National Institute of Aquatic Resources, Technical University of Denmark, Kemitorvet Bygning 202, DK-2800 Kongens Lyngby, Denmark; National Institute of Aquatic Resources, Technical University of Denmark, Kemitorvet Bygning 202, DK-2800 Kongens Lyngby, Denmark; National Institute of Aquatic Resources, Technical University of Denmark, Kemitorvet Bygning 202, DK-2800 Kongens Lyngby, Denmark; Department of Biological Sciences, University of Bergen, Thormøhlensgate 53B, P.O. Box 7803, NO-5020 Bergen, Norway; Bjerknes Centre for Climate Research, Jahnebakken 5, P.O. Box 7800, NO-5020 Bergen, Norway; Norwegian Institute for Nature Research, Thormøhlensgate 55, NO-5006 Bergen, Norway

**Keywords:** oil spill, climate change, Disko bay, hatching success, nauplii size, Arctic

## Abstract

The rise in shipping due to the reduction of sea ice in the Arctic is expected to increase oil spill incidents. *Calanus hyperboreus* is a key organism in the Arctic food web and has a complex life cycle including pronounced seasonality and wide vertical distribution. Reproduction and spawning take place at depth in late winter, and the eggs float toward the surface, where they may encounter brine release and oil at the interface between water and sea ice. In the laboratory, we exposed *C. hyperboreus* eggs and nauplii to crude oil (1 μL L^−1^) and high salinity (35.5 ppt), reflecting such conditions. Hatching success and nauplii size were not affected by exposure to oil alone, but decreased when exposed to high salinity or a combination of the two. The stressors did not impact the mortality of eggs and nauplii, which varied between 13.7% and 33.7% for the entire 6-day study period.

## INTRODUCTION

The large Arctic copepod *Calanus hyperboreus* diapause at depth and starts spawning during late winter (Conover, 1967; Hirche and Niehoff, 1996; Henriksen *et al*., 2012). Their eggs are mostly made up of lipids enclosed in a thin membrane of fatty acids (Conover, 1967; Hirche and Niehoff, 1996), making them positively buoyant. The eggs hatch ~4.3 days after spawning, and the first two nonfeeding naupliar stages survive on their lipid reserve, composed of energy-rich wax esters (Conover, 1967; Jung-Madsen *et al*., 2013). In shallow areas, the offspring will reach the sea ice-water interface as eggs or newly hatched nauplii, where they may be exposed to high salinities due to brine expulsion during sea ice formation (Cox and Weeks, 1974). Furthermore, increased shipping activities in the Arctic (Aksenov *et al*., 2017) will enhance the likelihood of oil spills (Wright *et al*., 2022). Toxic compounds in crude oil can be passively absorbed or passed via maternal transfer to the egg (Hansen *et al*., 2017; Toxværd *et al*., 2018). Even though the impacts of oil exposure are well described (Nørregaard *et al*., 2015; Agersted *et al*., 2018), the cumulative impacts of multiple stressors, including oil and high salinities associated with the brine, are unknown. Here, we hypothesize that exposure to crude oil and high salinity will jointly reduce hatching success and naupliar size of *C. hyperboreus*.

## METHOD

*C. hyperboreus* females were collected using a 300 μm WP3 net in the upper 50 m of the water column (total depth ~100 m), outside Qaqqaliaq (69.2472° N, 53.590467° W), Qeqertarsuaq, Disko Bay, West Greenland on the 22nd of March 2023. In the laboratory, the sampled copepods were transferred to *in situ* seawater (33 ppt, filtered through a 63 μm sieve) and kept at 0°C in a temperature-controlled room in the dark. Three females with ripe ovaries were sorted under a dissecting microscope on ice-chilled Petri dishes and transferred to a false-bottom cylinder (400 μm mesh) inside a 5 L bucket with filtered seawater. Females were moved to new buckets every 12 h to ensure that the eggs collected were of approximately the same age.

We used five treatment groups, four replicates (bottles) within each group, and 20 eggs (from the same clutch) per replicate (Table IA). The two stressors were high salinity and oil exposure. Eggs in treatments with 33 ppt remained in 1 L of seawater throughout the experiment from day one. High salinity treatments were kept in 1 L bottles filled halfway (500 mL) with 33 ppt seawater for 4 days (estimated time until hatching), after which salinity was raised to 35.5 ppt by adding 500 mL of 38 ppt seawater, prepared separately by adding 24 g aquarium salt to 5 L of 33 ppt seawater (Table IA). Salinities were verified with a Leitz refractometer. To test the effect of increased salinity and crude oil on hatching success and newly hatched nauplii, we exposed the eggs to these conditions on day 4. This timing was chosen to mimic a scenario in which eggs, spawned at 50 m depth (where the females in this case were found), ascend at an average rate of 8 m per day (Jung-Madsen *et al*., 2013) and reach the surface approximately when hatching. At the surface, they could encounter higher salinity from brine release and potential crude oil pollution. Crude oil (Light Louisiana Sweet, 1 μL L^−1^) prepared via high-speed magnetic stirring, following Almeda *et al*. (2021), was added to designated treatments on the specified days (Table IA). The chemical composition of the exposure water is given in Rist *et al*. (2024). Parallel to the 1 L bottles, identical treatments and replicates were prepared in 20 mL glass vials to facilitate daily inspection under a stereo microscope, while minimizing handling in the 1 L bottles of the main experiment. No results were yielded from the small vials. All the bottles were kept at 0°C in a temperature-controlled room in the dark. The total duration of the experiment was 6 days (time of observation of naupliar stages NII in vials). The main experiment was terminated on day 6 by adding 2 mL Lugol. Nauplii were counted, and carapace length was measured under an Olympus SZ40 Stereo Microscope. A minimum of 10 nauplii from each replicate were measured, or all if fewer were present. Mean nauplii size was calculated from all individuals of stages NI and NII. Mortality was calculated on day six based on the number of missing or damaged eggs or nauplii.

**Table I:** (A) Experimental setup, where n is the number of bottles per treatment and N is the number of eggs per bottle. S_33_ = salinity of 33 ppt, S_35.5_ = salinity of 35.5 ppt, O_0_ = no oil, O_1_ = 1 μL L^−1^ of crude oil. The superscripts ^1^ and ^4^ indicate the day when exposure to higher salinity, oil, or both started. The eggs in treatments S_35.5_O_0_^4^ and S_35.5_O_1_^4^ were kept in 33 ppt seawater until they were exposed to higher salinity on day 4. All treatments were kept at 0°C. (B) Measurements and standard deviation of hatching success, mean nauplii size (both stages NI & NII), and mortality for the different treatments. The number of individuals measured for the mean nauplii size is given in parentheses

	Treatment		S_33_O_0_^1^	S_33_O_1_^1^	S_33_O_1_^4^	S_35.5_O_0_^4^	S_35.5_O_1_^4^
		*n*	4	4	4	4	4
		*N*	20	20	20	20	20
A	Day of exposure		1	1	4	4	4
	Salinity (ppt)		33	33	33	(33) 35.5	(33) 35.5
	Crude oil (μL⋅L^−1^)		0	1	1	0	1
	Duration (days)		6	6	6	6	6
B	Hatching success		0.787 ± 0.201	0.737 ± 0.170	0.800 ± 0.108	0.687 ± 0.143	0.487 ± 0.165
	Nauplii size (μm)		237.1 ± 11.9(40)	238.2 ± 12.1(40)	234.6 ± 12.3(40)	213.4 ± 8.3(30)	213.5 ± 9.5(26)
	Mortality		0.162 ± 0.201	0.200 ± 0.158	0.137 ± 0.125	0.237 ± 0.094	0.337 ± 0.232

To test for differences in hatching success and mortality between treatments (i.e. proportions between 0 and 1) we used a beta regression model and estimated marginal means with pairwise comparisons from the “betareg” package (Cribari-Neto and Zeileis, 2010). Nauplii size was tested for normality (Shapiro–Wilk) and homogeneity of variance (Bartlett) (see Supplementary Table 1). Differences in nauplii size were tested with Kruskal–Wallis’s and pairwise Wilcoxon tests. All analyses and figures were conducted using R version 4.2.2 (R Core Team, 2021).

## RESULTS

**Fig. 1:**
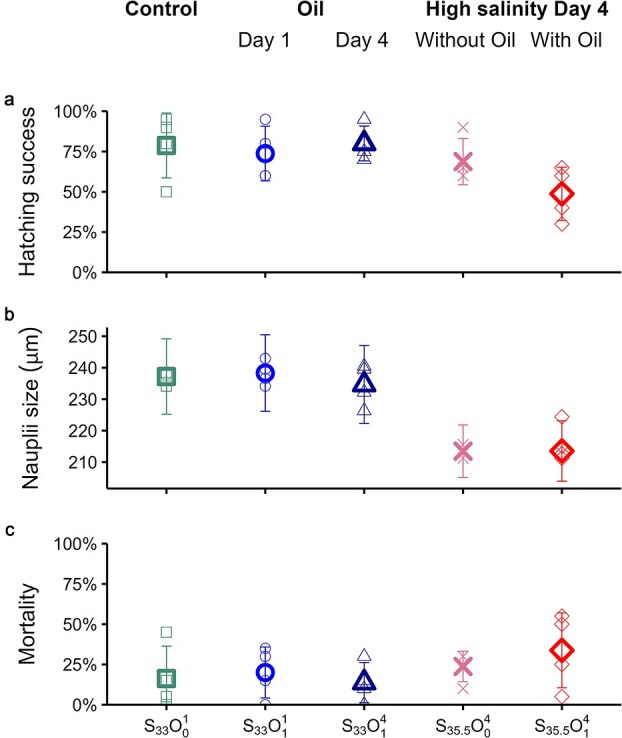
Impact of crude oil and high salinity on egg hatching success (a), mean nauplii size (b), and egg and nauplii mortality (c) per treatment on day 6 of the experiment. S stands for salinity: 33 or 35.5 ppt, as indicated by the subscript. O stands for crude oil: 0 or 1 μL L^−1^ as the subscript indicates. The superscript for all treatments indicates the day of exposure to the stressors.

The average hatching success varied from 48.7 ± 16.5% to 80 ± 10.8% (Table IB, Fig. 1a). The treatment with high salinity and oil exposure on day 4 (S_35.5_O_1_^4^) had lower hatching success (*p* = 0.004), compared to the control (S_33_O_0_^1^-S_35.5_O_1_^4^, *p* = 0.017) and to the group exposed to oil only from day 4 (S_33_O_1_^4^-S_35.5_O_1_^4^, *p* = 0.023) (see Supplementary Tables 2–4). Mean nauplii size (Table IB; Fig. 1b) varied significantly between treatments (*p*< 0.001, see Supplementary Tables 7 and 8), with nauplii exposed to high salinity (S_35.5_O_0_^4^ and S_35.5_O_1_^4^) being 23.2 ± 3.5 μm (9.8%) smaller on average. Mean mortality varied between 13.7% and 33.7% for all treatments (Table IB; Fig. 1c), with no apparent differences between the groups (*p* = 0.27) (see Supplementary Tables 2, 5, and 6).

## DISCUSSION

The observed hatching success and nauplii size in the control correspond well with findings from other studies (Conover, 1967; Jung-Madsen *et al*., 2013). Our study provides some of the first quantified mortality estimates at early developmental stages for *C. hyperboreus*. The reduced nauplii size under high salinity could be due to osmotic stress diverting resources from growth (Péqueux, 1995). Contrary to our expectations, crude oil exposure showed no effects on size. Nørregaard *et al*. (2014) observed reduced hatching success when females were exposed to 100 nΜ (~20.2 μg⋅L^−1^) pyrene—a concentration much higher than in our study. The 1 μL L^−1^ (~0.84 ppm) crude oil used here more realistically approximates concentrations near oil spills (Mcauliffe *et al*., 1981). Hence, the lack of observed response in nauplii size may be due to the lower concentration or the uptake operating via the exposed mothers (Hansen *et al*., 2017). Interestingly, hatching success declined under combined high salinity and crude oil, which could be an indication of a cumulative effect, consistent with studies reporting multiple stressor effects on other marine copepods (Almeda *et al*., 2021; Dinh *et al*., 2022; Makri *et al*., 2024; Rist *et al*., 2024).

## CONCLUSION

In conclusion, we provide a pilot study of how salinity and oil pollution impact vital rates of early life stages in a large and long-lived high-Arctic copepod. We document possible interactions and thereby multiple stressor effects. In the future, less and thinner sea ice and increased shipping and coastal activity may lead to more frequent ice openings and increased icebreaking, enhancing brine release into surface waters. Our findings show that high salinity significantly reduces nauplii size, while the combination of high salinity and crude oil further decreases hatching success in *C. hyperboreus*. Our study also points to the intricate relationships between environmental change and adaptations to seasonality, such as timing of reproduction and seasonal migrations (Varpe, 2017). We recommend that future studies for *C. hyperboreus* and related species expand on the life stages covered and on linkages between stressors and critical life-cycle stages and behaviors. Our study draws needed attention to the ecological impacts of a changing Arctic, where reduced copepod survival and growth may disrupt energy flow to higher trophic levels.

## Supplementary Material

Ntinou_et_al_2025_ChypEggExp_Suppl_fbaf053
