# Supplementary material for: Effects of crude oil and high salinity on eggs and early naupliar stages of the copepod Calanus hyperboreus
Source: J Plankton Res. 2025 Sep 28;47(5):fbaf053. doi: 10.1093/plankt/fbaf053 (PMC12476833; doi:10.1093/plankt/fbaf053)
Supplement: Ntinou_et_al_2025_ChypEggExp_Suppl_fbaf053 [file ntinou_et_al_2025_chypeggexp_suppl_fbaf053.docx]

Effects of crude oil and high salinity on eggs and early naupliar stages of the copepod *Calanus hyperboreus*

Iliana Vasiliki Ntinou^a, b,^ *, Sinja Rist^c^, Sofie Rask^c^, Martin Lindegren^c^, Torkel Gissel Nielsen^c^, Øystein Varpe^a, b, d^

^a^ Department of Biological Sciences, University of Bergen

^b^ Bjerknes Centre for Climate Research, Bergen, Norway

^c^ National Institute of Aquatic Resources, Technical University of Denmark, Kgs. Lyngby, Denmark

^d^ Norwegian Institute for Nature Research, Bergen, Norway

*Corresponding author. Email: [iliana.ntinou@uib.no](mailto:iliana.ntinou@uib.no), Postal Address: Thormøhlensgate 53B, Department of Biological Sciences University of Bergen, P.O. Box 7803, N-5020 Bergen Norway

**Supplementary material**

**Table 1.** Results of Shapiro-Wilk test for normality and Bartlett test for homogeneity of variances for nauplii size.

|  |  | Shapiro – Wilk test | |  | Bartlett’s test | | |
| --- | --- | --- | --- | --- | --- | --- | --- |
|  |  | Stat. | Sig. |  | Stat. | Df. | Sig. |
| Nauplii size |  | 0.968 | < 0.001 |  | 7.341 | 4 | 0.118 |

**Table 2.** Summary of beta regression model outputs for Hatching Success and Mortality, including coefficient estimates, randomized quantile residuals, precision model parameters (φ), and model statistics. Coefficients are presented for each treatment level relative to the reference group. P-values indicate the significance of each predictor.

|  | Hatching success | | | | | | | |  | | Mortality | | | | | | | | | | |
| --- | --- | --- | --- | --- | --- | --- | --- | --- | --- | --- | --- | --- | --- | --- | --- | --- | --- | --- | --- | --- | --- |
| **Coefficients** | Estimate | | Std Error | | z-value | | Pr(>\|z\|) | |  | Estimate | | | | | Std Error | | z-value | | | Pr(>\|z\|) | |
| S_33_O_0_^1^ | 1.386 | | 0.381 | | 3.634 | | < 0.001 | |  | -1.002 | | | | | 0.539 | | -1.858 | | | 0.063 | |
| S_33_O_1_^1^ | -0.275 | | 0.519 | | -0.531 | | 0.595 | |  | 0.128 | | | | | 0.458 | | 0.280 | | | 0.779 | |
| S_33_O_1_^4^ | -0.048 | | 0.529 | | -0.092 | | 0.926 | |  | -0.172 | | | | | 0.470 | | -0.365 | | | 0.714 | |
| S_35.5_O_0_^4^ | -0.592 | | 0.508 | | -1.163 | | 0.244 | |  | 0.221 | | | | | 0.492 | | 0.450 | | | 0.659 | |
| S_35.5_O_1_^4^ | -1.437 | | 0.499 | | -2.875 | | 0.004 | |  | 0.621 | | | | | 0.467 | | 1.329 | | | 0.183 | |
| **Residuals** | Min | 1Q | | Median | | 3Q | | Max |  | Min | | | | 1Q | | Median | | | 3Q | | Max |
|  | -1.923 | -0.065 | | -0.250 | | 0.738 | | 1.751 |  | -1.612 | | | | -0.688 | | 0.034 | | | 0.718 | | 1.913 |
| **Precision model with identity link** | **Term** | **Estimate** | | **Std Error** | | **z-value** | | **Pr(>\|z\|)** |  | **Term** | | **Estimate** | | | | **Std Error** | | **z-value** | | | **Pr(>\|z\|)** |
|  | phi | 8.541 | | 2.608 | | 3.276 | | 0.001 |  | phi | | | 21.39 | | | 19.77 | | | 1.081 | | 0.279 |
| **Model statistics** | **Type of estimator** | | **Log-likelihood** | | **Pseudo R-squared** | | **Iterations** | |  | **Type of estimator** | | | | | **Log-likelihood** | | **Pseudo R-squared** | | | **Iterations** | |
|  | Maximum Likelihood | | 12.1 on 6 Df | | 0.331 | | 14 (BFGS) + 2 (Fisher scoring) | |  | Maximum Likelihood | | | | | 3.186 on 7 Df | | 0.331 | | | 68 (BFGS) | |

**Table 3.** Estimated marginal means of hatching success for each treatment group with 95% confidence intervals.

| **Treatment** | **emmean** | **SE** | **df** | **asymp.LCL** | **asymp.UCL** |
| --- | --- | --- | --- | --- | --- |
| *S_33_O_0_^1^* | 0.800 | 0.061 | Inf | 0.680 | 0.920 |
| *S_33_O_1_^1^* | 0.752 | 0.067 | Inf | 0.620 | 0.884 |
| *S_33_O_1_^4^* | 0.792 | 0.062 | Inf | 0.670 | 0.914 |
| *S_35.5_O_0_^4^* | 0.689 | 0.073 | Inf | 0.545 | 0.833 |
| *S_35.5_O_1_^4^* | 0.487 | 0.080 | Inf | 0.329 | 0.645 |

**Table 4.** Tukey-adjusted pairwise comparisons of treatment groups based on estimated marginal means of hatching success.

| **Contrast** | **Estimate** | **SE** | **df** | **z.ratio** | **Pr(>\|z\|)** |
| --- | --- | --- | --- | --- | --- |
| *S_33_O_0_^1^ - S_33_O_1_^1^* | 0.047 | 0.089 | Inf | 0.531 | 0.984 |
| *S_33_O_0_^1^ - S_33_O_1_^4^* | 0.007 | 0.086 | Inf | 0.092 | 1.000 |
| *S_33_O_0_^1^ -S_35.5_O_0_^4^* | 0.111 | 0.094 | Inf | 1.174 | 0.766 |
| *S_33_O_0_^1^ - S_35.5_O_1_^4^* | 0.312 | 0.101 | Inf | 3.092 | 0.017 |
| *S_33_O_1_^1^ - S_33_O_1_^4^* | -0.039 | 0.090 | Inf | -0.439 | 0.992 |
| *S_33_O_1_^1^ - S_35.5_O_0_^4^* | 0.063 | 0.099 | Inf | 0.641 | 0.968 |
| *S_33_O_1_^1^ - S_35.5_O_1_^4^* | 0.264 | 0.105 | Inf | 2.522 | 0.085 |
| *S_33_O_1_^4^ - S_35.5_O_0_^4^* | 0.103 | 0.095 | Inf | 1.081 | 0.816 |
| *S_33_O_1_^4^ - S_35.5_O_1_^4^* | 0.304 | 0.102 | Inf | 2.993 | 0.023 |
| *S_35.5_O_0_^4^ - S_35.5_O_1_^4^* | 0.201 | 0.109 | Inf | 1.847 | 0.346 |

**Table 5.** Estimated marginal means of mortality for each treatment group with 95% confidence intervals.

| **Treatment** | **emmean** | **SE** | **df** | **asymp.LCL** | **asymp.UCL** |
| --- | --- | --- | --- | --- | --- |
| *S_33_O_0_^1^* | -1.003 | 0.540 | Inf | -2.061 | 0.055 |
| *S_33_O_1_^1^* | -0.874 | 0.367 | Inf | -1.594 | -0.155 |
| *S_33_O_1_^4^* | -1.175 | 0.429 | Inf | -2.016 | -0.334 |
| *S_35.5_O_0_^4^* | -0.781 | 0.302 | Inf | -1.374 | -0.189 |
| *S_35.5_O_1_^4^* | -0.381 | 0.298 | Inf | -0.965 | 0.203 |

**Table 6.** Tukey-adjusted pairwise comparisons of treatment groups based on estimated marginal means of mortality.

| **Contrast** | **Estimate** | **SE** | **df** | **z.ratio** | **Pr(>\|z\|)** |
| --- | --- | --- | --- | --- | --- |
| *S_33_O_0_^1^ - S_33_O_1_^1^* | -0.128 | 0.458 | Inf | -0.280 | 0.998 |
| *S_33_O_0_^1^ - S_33_O_1_^4^* | 0.172 | 0.471 | Inf | 0.365 | 0.996 |
| *S_33_O_0_^1^ -S_35.5_O_0_^4^* | -0.221 | 0.493 | Inf | -0.450 | 0.991 |
| *S_33_O_0_^1^ - S_35.5_O_1_^4^* | -0.622 | 0.468 | Inf | -1.329 | 0.673 |
| *S_33_O_1_^1^ - S_33_O_1_^4^* | 0.300 | 0.420 | Inf | 0.716 | 0.953 |
| *S_33_O_1_^1^ - S_35.5_O_0_^4^* | -0.093 | 0.383 | Inf | -0.243 | 0.999 |
| *S_33_O_1_^1^ - S_35.5_O_1_^4^* | -0.493 | 0.366 | Inf | -1.349 | 0.660 |
| *S_33_O_1_^4^ - S_35.5_O_0_^4^* | -0.393 | 0.426 | Inf | -0.923 | 0.888 |
| *S_33_O_1_^4^ - S_35.5_O_1_^4^* | -0.794 | 0.409 | Inf | -1.940 | 0.295 |
| *S_35.5_O_0_^4^ - S_35.5_O_1_^4^* | -0.400 | 0.349 | Inf | -1.146 | 0.781 |

**Table 7.** Kruskal-Wallis rank sum test for nauplii size per treatment.

|  | Kruskal-Wallis test | |
| --- | --- | --- |
| Chi-squared | Df | p-value |
| 90.407 | 4 | < 0.001 |

**Table 8.** Pairwise comparisons using the Wilcoxon rank sum test with Bonferroni correction for the p-value for nauplii size between treatments.

|  | S_33_O_0_^1^ | S_33_O_1_^1^ | S_33_O_1_^4^ | S_35.5_O_0_^4^ |
| --- | --- | --- | --- | --- |
| S_33_O_1_^1^ | 0.68 | - | - | - |
| S_33_O_1_^4^ | 0.54 | 0.33 | - | - |
| S_35.5_O_0_^4^ | < 0.001 | < 0.001 | < 0.001 | - |
| S_35.5_O_1_^4^ | < 0.001 | < 0.001 | < 0.001 | 0.68 |
